# Repositioning of HMG-CoA Reductase Inhibitors as Adjuvants in the Modulation of Efflux Pump-Mediated Bacterial and Tumor Resistance

**DOI:** 10.3390/antibiotics12091468

**Published:** 2023-09-20

**Authors:** Zsuzsanna Schelz, Hiba F. Muddather, István Zupkó

**Affiliations:** Institute of Pharmacodynamics and Biopharmacy, Faculty of Pharmacy, University of Szeged, Eötvös u. 6, 6720 Szeged, Hungary; schelz.zsuzsanna@szte.hu (Z.S.); hiba.161991@hotmail.com (H.F.M.)

**Keywords:** drug repositioning, efflux-mediated multidrug resistance, statins, HMG-CoA reductase inhibitors, mevalonate pathway, isoprenoid synthesis, reversal of multidrug resistance

## Abstract

Efflux pump (EP)-mediated multidrug resistance (MDR) seems ubiquitous in bacterial infections and neoplastic diseases. The diversity and lack of specificity of these efflux mechanisms raise a great obstacle in developing drugs that modulate efflux pumps. Since developing novel chemotherapeutic drugs requires large investments, drug repurposing offers a new approach that can provide alternatives as adjuvants in treating resistant microbial infections and progressive cancerous diseases. Hydroxy-methyl-glutaryl coenzyme-A (HMG-CoA) reductase inhibitors, also known as statins, are promising agents in this respect. Originally, statins were used in the therapy of dyslipidemia and for the prevention of cardiovascular diseases; however, extensive research has recently been performed to elucidate the functions of statins in bacterial infections and cancers. The mevalonate pathway is essential in the posttranslational modification of proteins related to vital eukaryotic cell functions. In this article, a comparative review is given about the possible role of HMG-CoA reductase inhibitors in managing diseases of bacterial and neoplastic origin. Molecular research and clinical studies have proven the justification of statins in this field. Further well-designed clinical trials are urged to clarify the significance of the contribution of statins to the lower risk of disease progression in bacterial infections and cancerous diseases.

## 1. Introduction

Hydroxy-methyl-glutaryl coenzyme-A (HMG-CoA) reductase inhibitors, also known as statins, are drugs used to treat dyslipidemia. The mechanism of action is based on inhibiting cholesterol synthesis in the hepatocytes. The lower cholesterol output results in lower circulating atherogenic lipid levels and a reduced risk of cardiovascular diseases, allowing statins to be commonly used to prevent cardiovascular pathologies [1,2]. Statins are the most extensively used and investigated lipid-modifying agents; therefore, a robust amount of data might show an evident relation between the beneficial effects other than lipid-lowering properties [3]. These pleiotropic effects include anti-inflammatory, renovascular-protective effects, prevention of thromboembolic events, and neuroprotective activities [4]. Commonly used statins include atorvastatin, simvastatin, rosuvastatin, and pravastatin. Statins’ side effects are generally mild and well tolerated. However, statins rarely cause life-threatening muscle toxicity (rhabdomyolysis), which could be prevented by closely monitoring the patients during statin therapy.

**Figure 1:**
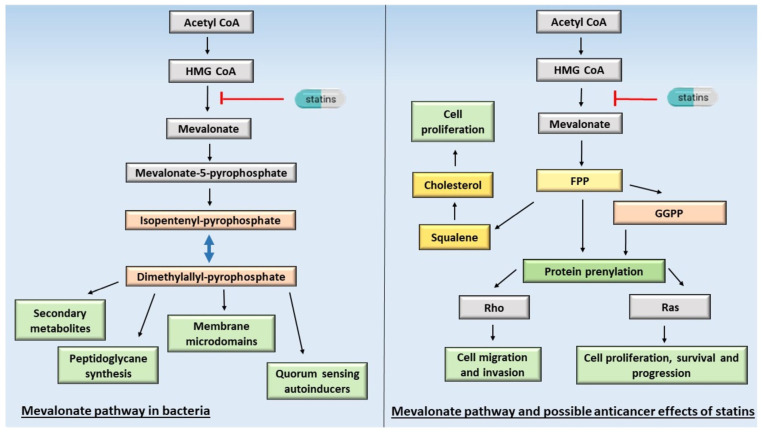
Mevalonate pathway in bacteria and eukaryotic cells, and possible targets of statin action. FPP, farnesyl-pyrophosphate; GGPP, geranylgeranyl-pyrophosphate.

The mevalonate pathway in humans is present in cells other than hepatocytes; therefore, the effect of statins can be observed in various cell types. Isoprenoids are diverse secondary metabolites of eukaryotic organisms. In fungi, isoprenoid intermediates like geranylgeranyl-pyrophosphate (GGPP) are essential for synthesizing antibiotics. In plants, it is a primary constituent in taxol and artemisinin biosynthesis [5]. In human cells, the mevalonate pathway is responsible for cholesterol biosynthesis and isoprenoid precursor production, e.g., the formation of farnesyl-PP (FPP), geranyl-PP (GPP), and GGPP. These intermediates are essential in the posttranslational modification of proteins involved in cancer progression and metastasis [6,7]. The synthetic pathways of isoprenoid intermediates occur widely in the kingdom of unicellular prokaryotic and the domain of eukaryotic organisms [8]. The mevalonate pathway in bacteria and tumor cells is shown in Figure 1.

The emergence of antibiotic resistance could be well recognized immediately after the introduction of the drug group, and an immense therapeutical burden has developed in a couple of decades, which necessitated profound research to give rise to antibiotics with higher selectivity and specificity. Plenty of attempts have been made to overcome antibiotic resistance, but it still poses enormous challenges to science [9,10]. Utilizing approved old drugs with new indications could be an applicable option to fight bacterial resistance. Several drug groups originally used in pathologies other than infectious diseases are potential agents to block the molecular steps of bacterial antibiotic resistance, e.g., antidepressants and neuroleptics [11,12,13]. The conventional strategies in antibiotic development are doubtful to keep up with the acquired resistance of bacterial pathogens. They often cannot provide a reasonable cost–benefit ratio during drug development. The success rate of receiving regulatory approval for a new antibiotic is very low, even after investing enormous financial and intellectual costs [14].

On the other hand, cancer is still one of the leading causes of death globally and is the second leading cause of death after cardiovascular diseases in the USA [15,16]. Applying successful preventative and therapeutic options for cancer is urgently needed [17]. Advanced cancer detection and novel therapies have improved survival in some cancer types, but in others, scarcely any progress has been made [18,19].

Drug repurposing (also called drug repositioning) is using a drug in another indication than the one for which it was initially approved and marketed [20]. The drug repurposing approach has many advantages, including a faster drug development time and a lower risk of failure because of the already completed preclinical tests, safety assessment, and substantially lower costs [21,22]. Drug repositioning depends on two scientific bases: First, through human genome expression, some biological targets are common to some diseases. Second, through the concept of pleiotropic drugs [23]. The drug’s repurposing has been implemented as an alternative to treat numerous human diseases [24,25,26].

**Figure 2:**
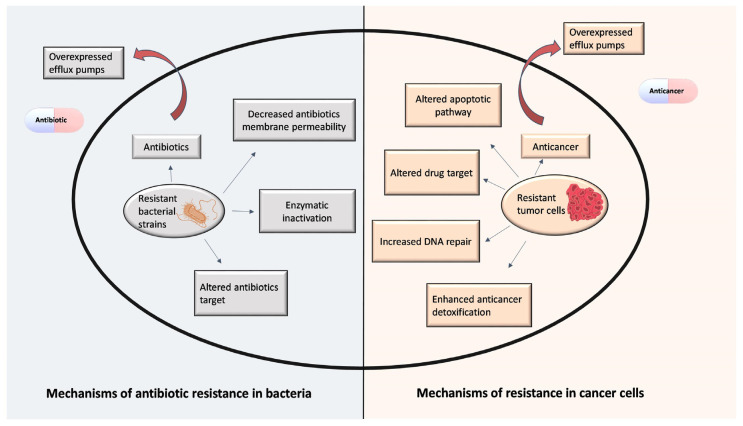
Differences and similarities between bacterial and cancer resistance.

Multidrug resistance (MDR) is a phenomenon in which resistance to one drug is accompanied by resistance to other drugs whose structures and mechanisms of action could be completely different. The term was first applied to the area of antibiotic-resistant bacteria [27] and was soon used in cancer chemotherapy [28]. The determination of mechanisms of MDR in bacteria and human cancer led to the development of therapeutic agents that may potentially overcome MDR [29,30]. Bacteria and tumor cells share similarities in efflux-mediated MDR [8]. Therefore, drug repurposing is a promising strategy to overcome drug resistance in bacterial infections and cancer. MDR mechanisms are summarized in Figure 2.

In the current review, we aim to provide an overview of the potential antibacterial and anticancer mechanisms of statins, the significance of statins in adjuvant bacterial and cancer therapy, and their efflux pump-modulating activity.

## 2. Antibacterial Effects of Statins

Since statins possess various pleiotropic effects in different pathologies, even in severe bacterial infections, it is a matter of great interest how these mechanistic gaps could be filled with in vitro and molecular investigations. In vitro antibacterial effects of statins were proven by determining MIC values, and a notable direct antibacterial effect was shown. The tested bacteria were selected based on the clinical relevance of the caused infectious diseases and the high rate of resistance occurring in the corresponding clinical isolates. *Staphylococcus aureus*, *Streptococcus pneumoniae*, *Pseudomonas aeruginosa*, *Haemophilus influenzae*, *Moraxella catarrhalis*, *Escherichia coli*, and *Enterococci* were investigated the most extensively [31,32,33,34,35]. The in vitro studies could show bacteriostatic and bactericidal effects but in a higher concentration range than the nanomolar concentrations appearing in the bloodstream during anti-dyslipidemic treatment. *S. pneumoniae* is a common causative agent in community-acquired pneumonia, but the emerging resistant variants and vaccination cannot guarantee the prevention of all streptococcal serotypes, which urges new approaches. Statins could have demonstrated protective effects in the airway epithelial cells against the streptococcal pore-forming toxin independently from the inhibitory effects on the mevalonate production [36]. It was described that bacteria can induce an immune response in the host by stimulating the synthesis of intermediates of the endogenous mevalonate pathway. The stimulatory effects were shown in the case of *E. coli* and *S. aureus* infections [37].

Statins have also been tested in animal models. *Klebsiella*-infected mice were pretreated with atorvastatin, and a better survival rate was determined with the co-administration of imipenem [38]. These results might support the possible beneficial outcome of systemic infections with the combination of statins and conventional antibiotics [38]. In several clinical observational studies, statins showed protective effects, e.g., in community-acquired pneumonia or tuberculosis (TB) [39,40,41,42]. In *K. pneumoniae* bloodstream infections, the mortality rate decreased by prior statin use [43]. There are ongoing clinical trials for assessing the protective effects of statins in the treatment of TB since emerging evidence shows a higher susceptibility to TB antibacterial treatment in patients under statin medication. In vitro and murine models for human TB revealed the beneficial effects of simvastatin by inducing host protection against TB with increasing phagosomal maturation and autophagy [44,45].

Clinical and epidemiological data prove that concomitant use of statins with antibiotic treatment provides a better prognosis in systemic bacterial infections or pneumonia [46]. Meta-analyses were undertaken to justify the relationship between statin use and a better outcome for septic patients [47]. Based on observational studies and clinical trials, it was concluded that statins exert beneficial effects in bacteriemia and severe infections, but the pieces of evidence are more robust in the observational studies; therefore, prospective clinical trials are needed to have a more conclusive therapeutical protocol design for the adjuvant application of HMG-CoA reductase inhibitors [48].

### 2.1. The Role of HMG-CoA Reductase in Bacteria

The mevalonate pathway is a metabolic pathway that is essential for the synthesis of bacterial metabolites, such as cholesterol, ubiquinone, and carotenoids, in both eukaryotes and prokaryotes and for peptidoglycan synthesis in bacteria [37,49,50]. In bacteria, the mevalonate pathway starts with the condensation of acetyl-CoA and acetoacetyl-CoA, catalyzed by the enzyme HMG-CoA synthase. The resulting HMG-CoA is then converted to mevalonate by HMG-CoA reductase, a key enzyme in the mevalonate pathway [51]. There are two major types of HMG-CoA reductases in bacteria that can be distinguished by their amino acid sequences. Class I. is the eukaryotic type, class II. is considered to be the archaeal and bacterial type. Class I. type, however, was also isolated from members of *Acinetobacter* and *Vibrio* strains. In class II., the enzyme was first characterized from *S. aureus* and later identified in different bacterial isolates (*Borrelia burgdorferi*, *Streptococcus pyogenes*, *Enterococcus faecalis*, and *Listeria monocytogenes*) [51]. Isoprenoid synthesis is essential in bacterial physiology, and pathogenesis and can determine whether a strain can grow under aerobic or anaerobic circumstances [52]. Many virulence factors in bacteria, such as toxins, adhesins, and siderophores, are synthesized with the contribution of this pathway. Moreover, the pathway is also involved in regulating membrane fluidity and cell wall biosynthesis, which are important factors for bacterial survival and virulence [53,54,55]. In addition to its role in bacterial physiology, the mevalonate pathway has also been implicated in developing antibiotic resistance. One of the major mechanisms of resistance is the efflux of antibiotics from the bacterial cell. The efflux pumps are membrane transporters that pump antibiotics out of the cell, thereby reducing their intracellular concentration and preventing their action [56]. It has been shown that the functions of efflux pumps are connected to the presence of isoprenoid intermediates and secondary metabolites. Statins might modulate the functions of efflux pumps in bacteria, thus affecting their resistance to antibiotics; however, the affinity of statins to the bacterial enzyme is lower than to the human reductase. Studies have shown that treatment with statins increases the susceptibility of bacteria to a wide range of antibiotics, including β-lactams, fluoroquinolones, and aminoglycosides [57]. The exact mechanism by which statins might modulate efflux pump expression is not yet fully understood, but it is believed to involve the mevalonate pathway and the downstream synthesis of isoprenoids. Statins, which target the HMG-CoA reductase enzyme, have been shown to modulate the functions of efflux pumps in bacteria and increase their susceptibility to antibiotics. Further research is needed to fully clarify this interaction’s mechanisms and develop new strategies to combat bacterial resistance [58]. Intracellular bacteria exploit the host cell’s enzymes to functionalize their virulence factors. Host-mediated posttranslational modification can be essential for intracellular pathogens for anchoring bacterial proteins to the host cytoplasmic membrane [59,60].

### 2.2. Isoprenoid Intermediates in Bacteria

GGPP is an isoprenoid molecule that plays a key role in synthesizing peptidoglycan in bacteria. Peptidoglycan is a critical component of the bacterial cell wall, providing strength and rigidity to the cell and protection from osmotic stress. GGPP is a substrate for the enzyme undecaprenyl pyrophosphate synthase (UPP synthase), which converts it into undecaprenyl pyrophosphate (UPP) [61]. UPP serves as a lipid carrier molecule that shuttles the building blocks of peptidoglycan from the cytoplasm to the cell membrane’s outer surface, where they are incorporated into the growing peptidoglycan layer [62]. The importance of GGPP and the mevalonate pathway in peptidoglycan synthesis has led to the investigation of statins as potential antimicrobial agents. By inhibiting HMG-CoA reductase and blocking the production of mevalonate, statins can decrease the availability of isoprenoid intermediates and inhibit peptidoglycan synthesis, ultimately leading to bacterial growth inhibition or death [63]. Some studies suggest that statins can also modulate the expression of genes involved in beta-lactam resistance, further enhancing their antimicrobial efficacy against resistant bacteria [64].

Statins may have bacterial-targeted and host-based antibacterial effects [58,59]. Some studies have shown that statins may have antibacterial effects against various bacteria, including *S. aureus*, *S. pneumoniae*, and *E. coli* [32]. These effects are thought to be due to the ability of statins to disrupt bacterial cell functions and interfere with bacterial growth and reproduction [65]. Although the host-related pleiotropic effects are more convincing, a plethora of evidence has been published about the immunomodulatory effects of this drug group [46].

Statins are currently not recommended for the treatment of bacterial infections. They should not be used in place of standard antibiotic therapy, but these drugs might benefit a patient taking long-term dyslipidemic medication [66]. The mechanism of action of statins can significantly affect the development of specific inhibitors of Class II. HMG-CoA reductase enzyme [67].

In bacteria, HMG-CoA reductase plays a similar role to its function in humans and other animals: it catalyzes a key step in the synthesis of isoprenoid compounds, which are essential building blocks for a wide range of cellular components, including cell walls, membranes, and electron transport chains [68]. Isoprenoids are also important for bacterial virulence and survival, as they are involved in the production of signaling molecules and the modulation of host immune responses. HMG-CoA reductase inhibitors can inhibit the activity of bacterial HMG-CoA reductase and thus interfere with isoprenoid biosynthesis, which may contribute to their antibacterial effects [69]. However, it is important to emphasize that the role of bacterial HMG-CoA reductase in isoprenoid synthesis is complex and varies between bacterial species [51,53]. Some bacteria have alternative pathways for isoprenoid synthesis that do not involve HMG-CoA reductase MEPK, and some may be able to compensate for the loss of isoprenoid biosynthesis by upregulating alternative pathways or by acquiring isoprenoid compounds from their environment [51]. Exogenous isoprenoids have been found to play a role in the functions of efflux pumps in some bacterial species [70]. However, some endogenous isoprenoids have been shown to modulate the activity of efflux pumps by binding to regulatory proteins and altering their expression or activity [71]. Lipid rafts that contain isoprenoids in the bacterial membranes are essential to the appropriate function of membrane proteins like efflux pumps and can affect the ability of the bacteria to pump out antibiotics and other compounds and contribute to antibiotic resistance [72]. Additionally, some efflux pumps are involved in the transport of isoprenoid compounds across the bacterial cell membrane [73]. For example, some bacteria use efflux pumps to export the isoprenoid quinone component menaquinone, which is involved in electron transport and energy production [74]. Disrupting the activity of efflux pumps can, therefore, influence the transport and metabolism of isoprenoid compounds in bacteria [61]. The relationship between isoprenoids and efflux pumps in bacteria is complex, multifaceted, and it varies between bacterial species and environmental conditions. Isoprenoid quinones like ubiquinone are present in the bacterial membrane and are responsible for membrane fluidity and stability, thereby providing osmoprotection [75]. Ubiquinone plays an essential role in the electron transport chain in *E. coli*. The synthesis is initiated by producing a benzoquinone head group, and a polyprenyl side chain is attached. This side chain is the product of the bacterial mevalonate pathway [76].

Bacterial membranes contain lipid rafts called functional membrane microdomains (FMMs) that are rich in isoprenoid molecules. These FMMs assemble proteins related to bacterial signaling and secretion [73,77]. The integrity of these microdomains is essential to the proper function of the membrane-associated proteins. Isoprenoid derivatives are part of these FMMs and belong to the minor membrane lipids. Disruption of these lipid rafts by inhibiting isoprenoid synthesis seems to be a relatively new approach to overcoming biofilm-associated resistance since key physiological functions of the bacterial cells are based on the presence of isoprenoid constituents in the microdomains. Mevastatin and lovastatin were reported to compromise the squalene synthesis of *S. aureus* because in Gram-negative bacteria, HMG-CoA reductase is a key enzyme in isoprenoid biosynthesis [78].

### 2.3. The Role of Bacterial Efflux Mechanisms in Antibiotic Resistance and the Effects of Statins on Antibiotic Resistance

Bacterial transporters are proteins involved in transporting molecules and substances across the bacterial cell membrane. They are essential for bacterial survival and play a key role in the uptake of nutrients and the efflux of toxic chemicals [56,79,80]. Seven different efflux pump systems can be distinguished in bacteria: the ATP-binding cassette (ABC) superfamily; the small multidrug resistance (SMR) superfamily; the multidrug and toxic compound extrusion (MATE) superfamily; the major facilitator superfamily (MFS); the resistance nodulation and cell division (RND) superfamily; the proteobacterial antimicrobial compound efflux (PACE) superfamily; and the p-aminobenzoyl-glutamate transporter family (AbgT) [81].

The structures and organization of efflux proteins differ in Gram-positive and Gram-negative bacteria. The mutation of efflux pumps’ regulatory genes leads to the efflux proteins’ overexpression, thereby resulting in a multidrug-resistant phenotype [82,83]. The reversal of bacterial resistance by efflux pump inhibitors might be an efficient means of suppressing antibiotic resistance.

Some studies have shown that statins can inhibit growth and resistance, increasing the susceptibility of bacteria to antibiotics [65]. These findings suggest that statins could potentially be used as adjuvants to enhance the effectiveness of antibiotics in treating bacterial infections [84]. There is a lack of direct scientific evidence for the efflux’s inhibitory effect of statins; however, compromising efflux functions by inhibiting isoprenoid intermediates might theoretically enhance the activity of the co-administered antibiotics, thereby leading to improved bacterial killing.

The susceptibility of different bacterial strains to statin treatment may vary depending on several factors, including the bacterial species, the type and concentration of statin used, and the mode of action of the statin [65]. Some studies suggest that Gram-positive bacteria, such as *S. aureus* and *S. pneumoniae*, may be more susceptible to statin treatment than Gram-negative bacteria [51]. Isoprenoids are involved in a variety of cellular processes, including cell wall biosynthesis, protein modification, energy metabolism, and stress responses. In Gram-positive bacteria, isoprenoids are synthesized by the mevalonate pathway, which is the target of statin drugs. Isoprenoids such as menaquinone, a component of the electron transport chain, and heme, a cofactor for many enzymes, are essential for the survival of Gram-positive bacteria [85]. Additionally, some Gram-positive bacteria use isoprenoids to produce virulence factors that contribute to their pathogenicity. Isoprenoids are involved in the biosynthesis of several virulence factors in Gram-positive bacteria. For example, isoprenoids are required for the production of lipoteichoic acids (LTAs), which are important components of the cell wall and are involved in the colonization of host tissues by bacteria such as *S. aureus* and *S. pneumoniae* or *Mycobacterium tuberculosis* [86,87,88]. Isoprenoids are also involved in the activity of exotoxins, such as pneumolysin in *S. pneumoniae*, which are inevitable virulence factors that contribute to bacterial pathogenesis by damaging host cells and tissues. Additionally, isoprenoids are involved in synthesizing quinolone molecules that act as signaling molecules and are essential for communication between bacteria and host cells during an infection [89].

Isoprenoids might play a role in the regulation of these efflux systems in an indirect way. The effects on membrane fluidity, membrane microdomains, and isoprenoid modification of important proteins related to bacterial resistance might be behind the possible modulatory effects of statins on efflux-mediated antibiotic resistance.

### 2.4. Quorum Sensing: A Possible Target for the Reversal of Efflux-Mediated Resistance

Quorum sensing is a bacterial cell-to-cell communication process that involves the production, detection, and response of small signaling molecules called autoinducers. This population-based behavior provides versatile advantages for the bacteria as a community and is crucial in regulating various bacterial characteristics, including virulence factor production, antibiotic resistance, and biofilm formation [90,91,92]. Biofilm formation is an ideal environment for pathogenic bacteria to develop multidrug-resistant phenotypes [93,94]. Recent studies have suggested that statins may impact quorum sensing in bacteria. The researchers found that atorvastatin inhibited quorum-sensing autoinducers’ production and reduced virulence gene expression in *P. aeruginosa*, leading to decreased bacterial virulence [95,96,97]. Atorvastatin and simvastatin exerted in vitro antibacterial and quorum-sensing inhibitory effects on plant and human pathogenic bacteria [98]. A novel study demonstrated that simvastatin and lovastatin could interfere with the quorum-sensing system of the human pathogen, *Bacillus subtilis* [99]. It was found that simvastatin reduced the expression of quorum-sensing-regulated genes and decreased biofilm formation in *L. monocytogenes*, suggesting that it could be used as an adjunct therapy to control *Listeria* infections. Statins may have potential as a new class of compounds for developing anti-virulence agents that target bacterial quorum-sensing systems. However, further research is needed to fully understand the mechanisms underlying the effects of statins on quorum sensing in bacteria [100].

**Figure 3:**
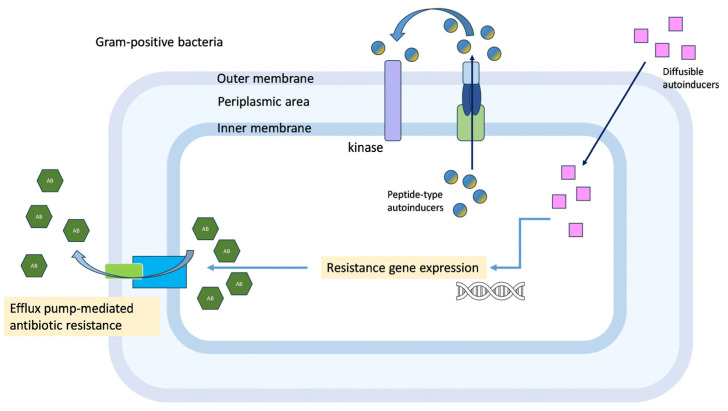
Quorum-sensing-regulated resistance mechanisms in Gram-positive bacteria.

Efflux systems and quorum sensing are both mechanisms that bacteria use to regulate their responses to external stimuli, including the presence of antibiotics or other environmental stressors. The growth rate and gene expression profiles of these bacteria are different from their planktonic counterparts. Bacteria in the biofilm are embedded within a matrix built up of polymeric macromolecules, and the behavior of these bacteria is regulated in a population-density-based manner. Quorum sensing and efflux pumps can be interconnected (Figure 3). Efflux systems can play a role in the secretion of quorum-sensing signals, which allows bacteria to communicate with each other and coordinate their responses to environmental changes [101]. Additionally, some efflux pumps are regulated by quorum sensing, meaning that the presence of quorum-sensing signals can modulate the expression and activity of the efflux pump. This mechanism can have important implications for the development of antibiotic resistance, as the coordinated action of both efflux systems and quorum sensing can lead to increased resistance and persistence of bacterial infections [102]. The inhibition of efflux pumps may lead to altered biofilm formation, forming a less resistant bacterial community.

The inhibition of quorum sensing by statins has reduced virulence and biofilm formation in various bacterial species, including *P. aeruginosa* and *S. aureus* [95]. The biofilm formation capacity of *S. aureus* was inhibited by rosuvastatin in combination with levofloxacin in topical formulations for wound infections caused by resistant bacterial strains [103,104].

### 2.5. Statins in Managing Specific Bacterial Infections

*Mycobacterium tuberculosis* poses a great obstacle in antibiotic therapy through the evolutionary development of multidrug-resistant strains, especially in immunocompromised patients [105]. Due to the adverse effects of long-term combinational antibiotic treatment, it would be favorable to shorten the chemotherapeutic regimen by applying adjuvants that enhance the effectiveness of the present treatment protocols. Statins are good candidates for that purpose in the host-directed approach [106]. Various mechanisms were clarified in the background of the host-directed approach. In vitro models have proven the higher resistance of macrophages against *M. tuberculosis* in the presence of statins, and the host may also develop a more robust immune response due to the statin treatment by promoting autophagy, cellular, and humoral immunology [44]. These findings are indirectly proven by in vitro results that indicated the effectivity of mevalonate pathway products (cholesterol, GGPP) to rescue statin-mediated antimycobacterial activity, which can also be observed in cancer cells [107]. In vivo murine models of chronic TB provided convincing evidence of statin’s effect and mechanism of action—the time to achieve TB-negative lungs under statin treatment shortened notably [108]. Statins were subjected to clinical trials based on their suspected in vivo effectivity in TB patients, and these trials showed that statin therapy may decrease TB incidence among diabetic and non-diabetic patients [109,110].

*S. aureus* infections are often in the background of bacteriemia. In cohort studies, the concomitant use of statins strengthened the favorable outcome of the antimicrobial therapy, and a significant decrease in the 30-day mortality rate could be observed [111]. Systemic *Staphyloccus* infections were investigated in different clinical study designs to confirm supportive statin effects, and most of the trials could denote the adjuvant impact of these drugs to enhance the host immune response [63,112,113].

## 3. Anticancer Effects of Statins

Statins, as a repurposed anticancer drug, have received a lot of research interest since the 1990s. Pre-clinical data suggest that statins in specific cancer types inhibit tumor growth and induce apoptosis [114,115,116,117,118]. Since statins are relatively well tolerated, inexpensive, less toxic than conventional chemotherapeutics drugs, and are available as generic drugs, they could be considered an immediate approach to overcome drug resistance mechanisms, such as the overexpression of multidrug efflux pumps.

### 3.1. Potential Anticancer Mechanisms of Action of Statins

Statins interfere with the mevalonate pathway through the inhibition of the enzyme HMG-CoA reductase, leading to a decrease in mevalonate, other isoprenoid intermediates (including GGPP, FPP, and isopentenyl-PP (IPP)), and downstream cholesterol biosynthesis. Consequently, the inhibition of posttranslational modification and many key proteins, such as small monomeric GTPases (e.g., Ras, Rho, Rac, or Rap), is required for several cellular functions (Figure 1) [6,119,120,121]. So far, the changes associated with Ras protein activity in cancer cells have been more extensively studied among proteins of the GTPase family. Mutations in Ras genes have been observed in approximately 20–30% of human cancers, leading to a loss of intrinsic GTPase activity [122,123]. These mutations result in the persistent activation of Ras, which, accordingly, leads to the uncontrolled proliferation of cells. It was revealed that the inhibition of farnesylation of mutated Ras inhibits its activity in cancer cells, indicating that the inhibition of farnesylation seems to offer a vital means of hampering cancer progression [124,125]. On the contrary, interfering with RhoA-mediated signaling pathways by preventing geranylgeranylation might affect other cellular processes that are important for cancer progression, such as migration, invasion, and apoptosis [126,127]. It is reported that statins’ inhibition of the mevalonate pathway prevents radiation resistance in head and neck cancers, demonstrating that the mevalonate pathway might be a promising target for overcoming resistance development [128].

Lipophilic statins can diffuse passively through cell plasma membranes, while hydrophilic statins often require an OATP1B transmembrane transporter, expressed mainly in the liver [129,130]. The study found that lipophilic statins have higher pro-apoptotic activity than hydrophilic statins, which could be explained by differential transmembrane uptake [131]. Due to these differences, lipophilic statins have a higher cytotoxic potential; while undesirable in dyslipidemia therapy, this might be advantageous in cancer treatment.

The additional antitumor effect of statins is facilitated through the inhibition of angiogenesis. The exact effects of statins on angiogenesis are controversial since both inhibition and stimulation have been illustrated. The pro- or anti-angiogenic effects of statins depend on the exposed cell type, the drug concentration, and the studied model [132,133,134]. Anti-angiogenic activity is achieved by suppressing cytokine-induced production of the pro-angiogenic factor vascular endothelial growth factor (VEGF) [134,135]. Moreover, statins can inhibit angiogenesis via the inhibition of endothelial cell proliferation and impede the adhesion of endothelial cells to the extracellular matrix [136].

Furthermore, statins reduce the metastatic potential of tumor cells [137,138] by preventing the expression of adhesion molecules such as E-selectin on endothelial cells [139]. These adhesion molecules are essential for attaching tumor cells to the endothelium, which is one of the first steps for metastases development. An additional mechanism includes inhibiting the synthesis of mediators inducing the migration of tumor cells to the bone [140]. In an in vitro study, statins prevent metastasis in renal endometrial cancer cells via the AKT/mTOR, ERK, and JAK2/STAT3 pathways [141]. Atorvastatin has been shown to inhibit endothelial mesenchymal transition (EMT), a possible mechanism for the statin-mediated suppression of metastasis [142].

Statins could exhibit their antitumor effects by mevalonate-independent mechanisms. A study found that the lovastatin–docosahexaenoic conjugate exhibited anti-proliferative effects on triple-negative breast cancer cells (MDA-MB-231 and MDA-MB-468) and induced apoptosis [143]. A recent study also suggested a different mechanism of action for atorvastatin anti-invasive proprieties in PTEN-positive prostate cancer cells, inhibiting the Akt pathway via P2 × 7 and EHBP1 signaling [144]. In both cases, mevalonate could not reverse the control conditions, confirming independent activity from the mevalonate pathway.

Statins have also been shown to promote immunomodulatory effects. Cholesterol-enriched tumor microenvironments and tumor-infiltrating CD8+ T cells are associated with highly expressed immune checkpoint proteins and enhanced T-cell exhaustion, resulting in the escape of tumor cells’ immune surveillance. Reducing cholesterol levels in CD8+ T cells restores T-cell antitumor activity [145]. Furthermore, it was reported that the depletion of intracellular isoprenoid statins promotes the potent activation of co-cultured IL2-primed NK cells via IL-18, IL-1β, and caspase-1 activation [146]. Another finding has highlighted that fluvastatin could impair perforin and granzyme release but not NK FasL- and TNF-α-mediated cytotoxicity [147]. Additional investigation of the immunomodulatory properties of statins will open fruitful areas of research with significant clinical implications.

In summary, numerous mechanisms have been proposed for statins to exert antitumor activity; however, this requires further clarification.

### 3.2. Statins in Epidemiologic Studies

Several observational population-based studies indicate that statins have potential anti-neoplastic properties (Table 1). A 15-year large-scale observational study of a Danish subpopulation indicated a reduction in cancer-related mortality among cancer patients using statins in 13 cancer types compared with patients who did not use statins [148]. A meta-analysis of 1,111,407 cancer patients revealed that statins were associated with improved overall survival and cancer-specific survival [149]. However, a large meta-analysis of 17 randomized studies and 25 observational studies reviewed the effect of statins on all cancer risk, suggesting no effect on overall incidence in the short term (relative risk (RR), 0.96; 95% CI, 0.72–1.2) [150]. A meta-analysis of breast cancer studies found that the general use of statins was associated with lower cancer-specific and all-cause mortality. In addition, lipophilic statins were associated with lower breast cancer-specific and all-cause mortality; the protective effect of hydrophilic statins was weak only against all-cause mortality [151]. Nevertheless, in another meta-analysis of 7858 breast cancer patients at about 5 years of mean follow-up periods, no correlation between statin use and breast cancer risk was detected (relative risk (RR), 1.02; 95% CI, 0.89–1.18) [152]. Regarding prostate cancer, a study of 4204 patients who underwent prostate biopsy proposed that statins users had a significantly reduced risk of prostate cancer compared with those who did not use statins (RR, 0.92; 95% CI, 0.85–0.98) [153]. In addition, it had been suggested that statins users have less frequent high-grade prostate cancer and lower prostate cancer volume. Another analysis of 1001 men with prostate cancer, where 289 were statin users, reported a 0.19 hazard ratio (HR) for prostate cancer-specific death among men who used statins compared to men who did not use statins (95% CI, 0.06–0.56). The study suggested possible mechanisms could be through statins reduction of mevalonate, circulating cholesterol, and cholesterol-rich domains in cell membranes, which play a role in intracellular signaling pathways related to prostate cancer cell survival [154]. On the other hand, the results of a population-based epidemiologic study of 1001 prostate cancer patients indicated that the use of statins was not associated with overall prostate cancer risk [155], while the study has some concerns, including the relatively small sample size and the potential selection and recall biases. An analysis of pancreatic ductal adenocarcinoma patients found that using statins was associated with improved patients’ overall survival [156]. Moreover, statins were associated with improved patient outcomes in 303 advanced adenocarcinoma pancreatic cancer patients receiving chemoradiation and surgery [157]. A study of colon cancer patients revealed that the use of statins after disease diagnosis was associated with a significantly lower risk of death from any cause (adjusted RR, 0.67; 95% CI, 0.51–0.87) and a lower risk of cancer-related death (adjusted RR, 0.66; 95% CI, 0.49–0.89). Notably, the effect of statin use was greater for patients with intact bone morphogenetic protein (BMP) signaling, independent of KRAS mutation status [158]. A recent meta-analysis of colorectal cancer (CRC) cases reported that statin use was significantly correlated with a reduction in overall mortality and cancer-specific mortality [159]. In gastric cancer, a cohort study showed that statins could be related to a reduction in gastric cancer mortality in the general population but have no association with the incidence of gastric cancer [160]. A meta-analysis of 59,073 hepatocellular carcinoma (HCC) patients showed that statin use was significantly associated with a reduced risk of HCC incidence compared to non-users of statins (RR, 0.54; 95% CI, 0.47–0.61) [161]. A recent prospective observational study of ovarian cancer patients suggested that the use of lipophilic statins was correlated with an increase in patient survival, but the effect was not observed in hydrophilic statins [162]. In cervical cancer, an HR of 0.83 for cervical cancer risk and statin use was reported (95% CI of 0.67–0.99). In addition, statin use was correlated with reduced total gynecological cancer mortality (HR, 0.70; 95% CI, 0.50–0.98) [163]. Another study showed that in cervical cancer patients, the lipophilic statin user had better outcomes compared with the non-user (progression-free survival: HR, 0.062; 95% CI, 0.008–0.517; overall survival: HR, 0.098; 95% CI, 0.041–0.459) [164].

The inconsistency in published epidemiological findings regarding statin use and cancer incidence and mortality could be due to differences in follow-up periods and other limitations in retrospective and observational study designs. A long enough follow-up period could be considered to detect the relationship between statin use and cancer development.

### 3.3. In Vitro Anticancer Effects of Statins

The antiproliferative effects of statins are powerfully demonstrated by in vitro studies on cancer cell lines (Table 2). The effect is based on both the inhibition of proliferation and the induction of apoptosis by modulating the expression and activity of several proteins involved in cell cycle progression. Statins through the depletion of isoprenoids lead to pro-apoptotic effects in a variety of cancer cells, including CRC cells [178], breast cancer [179,180], lung adenocarcinoma, glioblastoma cell lines (GBM) [179], ovarian [181], thyroid [182], cholangiocarcinoma [183], hematopoietic tumor cells [184], and murine melanoma cells [185]. Concerning cell cycle arrest, statins arrest the cell cycle at the G1 phase, as has been revealed for prostate cancer [186,187], breast cancer cells [185], multiple myeloma [188], and renal cell carcinoma (RCC) [189].

EMT, which is an effective mechanism of cancer metastasis, is a dynamic multi-gene programming cycle [190]. In breast cancer stem-like cells, lipophilic statins have been found to antagonize EMT signaling pathways by inhibiting the mevalonate pathway [191]. Evidence indicates that statins disrupting geranylgeranylation and farnesylation of small GTPases inhibit tumor metastasis [126,192]. For example, lipophilic statins reduce cell migration, invasion, and colony formation of metastatic prostate cancer cells [193], and attenuated rac1 phosphorylation of simvastatin reduces the invasion ability of RCC [194]. Further, a direct effect of lovastatin on tumor cell adhesion was reported in HUVEC cells treated with a combination of lovastatin and all-trans retinoic acid through the reduction of E-selectin expression [195].

Moreover, studies revealed that combining statins with conventional anticancer drugs in various cell lines improved therapeutic effects, as shown in breast cancer, hepatocellular carcinoma cancer, ovarian cancer, small-cell lung cancer, acute leukemia, and GBM [196,197,198,199,200,201,202]. A combination of statins and ionizing radiation has successfully suppressed lung tumors [203].

### 3.4. In Vivo Anticancer Effects of Statins

To verify the detected in vitro beneficial effects of statins on cancer cells, in vivo studies have been conducted (Table 2). Studies performed on mice xenografts confirmed the potential for statin-induced apoptosis in breast cancer [204], prostate cancer [205], and renal models [194]. Targeting of the MA pathway and intraperitoneal injection of pitavastatin resulted in the growth inhibition of subcutaneous glioblastoma tumor cells [206]. Simvastatin significantly inhibits tumor growth and bone metastasis, concomitantly reducing MAPK/ERK activity in a xenograft mouse lung cancer model [207]. Another investigation confirmed the association between monomeric GTPases and cancer metastasis, in which atorvastatin inhibited metastasis of RhoC-overexpressed melanoma cells in vivo [192].

Combined therapies of statins and current anticancer drugs have been subject to assessment in numerous in vivo models. In an in vivo GBM model, atorvastatin synergistically enhanced the efficacy of temozolomide to prevent tumor growth via Ras isoprenylation suppression and downstream Ras, ERK, rS6, and elF4E activation reduction [208]. Furthermore, administering low doses of a combination of atorvastatin and celecoxib in mice was more effective than each treatment and efficiently prevented prostate cancer progression from androgen-dependent to androgen-independent [209]. Gemcitabine and fluvastatin combination therapy in pancreatic cancer xenografts leads to suppression and a delay in tumor growth relapse [210]. Finally, simvastatin and cetuximab combination therapy in a murine model significantly reduced the proliferation of xenograft Kirsten rat sarcoma viral oncogene homolog (KRAS)-mutated CRC tumors compared to cetuximab alone [211].

**Table 2:** The anticancer effect of statins in preclinical studies.

Statins	Cancer Type	Study Type	Combination Agent	Findings	Ref.
Atorvastatin	Breast	In vitro	-	The antiproliferative effects of atorvastatin on breast cancer cells (MCF-7) are mediated by the induction of apoptosis and autophagy.	[212]
Simvastatin		In vitro	-	Simvastatin-induced breast cancer cell apoptosis, inhibited proliferation, and the deactivation of PI3K/Akt and MAPK/ERK pathways.	[213]
Simvastatin		In vitro	Doxorubicin	Simvastatin synergistically acts with the anticancer agent doxorubicin against breast cancer MCF-7 cells, probably through a down-regulation of the cell cycle or induction of apoptosis.	[214]
Lovastatin		In vitro	-	Lovastatin-mediated MCF-7 breast cancer cell death involves the activation of LKB1-AMPK-p38MAPK-p53-survivin cascade.	[215]
Mevastatin		In vitro and vivo	Histone deacetylase inhibitors (HDACi)	Combination treatment inhibited autophagic flux by preventing Vps34/Beclin 1 complex formation and downregulating prenylated Rab7, an active form of the small GTPase necessary for autophagosome–lysosome fusion in triple-negative breast cancer cells.	[216]
Lovastatin, mevastatin, pitavastatin calcium, and simvastatin		In vitro	-	Statins possess different anticancer activity in human breast cancer MDA-MB-231 and MCF-7 cell lines. Pitavastatin and simvastatin showed the highest activity in colony formation assay and migration and reduced the growth of MCF-7 spheroids.	[217]
Atorvastatin		In vitro/ex vivo and vivo	-	Statins can block the outgrowth of breast cancer metastases.	[218]
Atorvastatin	Prostate	In vitro	-	Atorvastatin induces autophagy in prostate cancer pC3 cells through the activation of LC3 transcription.	[219]
Simvastatin and fluvastatin		In vitro	-	Statins decrease cell proliferation and induce cell apoptosis, possibly mediated through the downregulation of AKT/FOXO1 phosphorylation in prostate cancer cells.	[220]
Atorvastatin, mevastatin, simvastatin and rosuvastatin		In vitro	-	Lipophilic statins reduce the migration and colony formation of PC-3 cells in human bone marrow stroma via inhibiting GGPP production, decreasing the formation, and the spread of metastatic prostate colonies.	[193]
Lovastatin	Ovarian	In vitro and vivo	-	Lovastatin influenced the expression of genes associated with DNA replication, glycolysis, Rho/PLC signaling, and cholesterol biosynthesis pathways.	[221]
Simvastatin, atorvastatin, rosuvastatin, lovastatin, fluvastatin, and pravastatin		In vitro	Carboplatin or paclitaxel	All the tested statins except pravastatin demonstrated single-agent activity against monolayers. Statins exhibited conflicting effects on the autophagy pathway.	[222]
Atorvastatin		In vitro	-	The antiproliferative activity of atorvastatin was connected with the induction of apoptosis, autophagy, cellular stress, and cell cycle G1 arrest through the inhibition of AKT/mTOR and the activation of the MAPK pathways. In addition, atorvastatin inhibited cell adhesion, invasion, and decreased the expression of VEGF and MMP 9. c-Myc was downregulated in ovarian cancer,	[223]
Atorvastatin, fluvastatin, and simvastatin	Cervical	In vitro	-	Statins exert antitumor effects on cervical cancer via the inhibition of cell proliferation and the induction of cell death and oxidative stress.	[224]
Simvastatin	Gastric	In vitro	-	Simvastatin inhibited the proliferation and migration of intestinal (NCI-N87) and diffuse (Hs746T) metastatic gastric tumor cell lines by reducing mevalonolactone, FPP, and GPP.	[225]
Simvastatin	Colorectal	In vitro	-	Simvastatin prompts the apoptosis of human colon cancer cells and inhibits IGF-1-induced ERK and Akt expression via the downregulation of IGF-1R expression and pro-apoptotic ERK activity.	[226]
Atorvastatin		In vitro and vivo	Nobiletin	Co-treatments of nobiletin and atorvastatin synergistically induced growth inhibitory effects, extensive cell cycle arrest, and apoptosis on the colon cancer cells. In addition, the combination synergistically enhanced chemopreventive activities against colon carcinogenesis in rats.	[227]
Atorvastatin	Liver	In vitro	-	Atorvastatin induces microRNA-145 expression in hepatic cancer cells HEPG2 through regulation of the PI3K/AKT signaling pathway.	[228]
Pravastatin and fluvastatin		In vitro	PBR ligands	Statins induced G1/S cell cycle arrest and apoptosis in hepatocellular carcinoma cells, and the efficacy of treatment with statins was synergistically enhanced by ligands of the peripheral benzodiazepine receptor (PBR).	[229]
Pitavastatin		In vitro and vivo	-	Pitavastatin inhibited growth and colony formation and induced arrest at the G1 phase of liver cancer Huh-7 and SMMC7721 cells. It also promoted caspase-9 and caspase-3 cleavage. Pitavastatin reduced tumor growth and improved the survival of tumor-bearing mice.	[230]
Simvastatin	Lung	In vitro and vivo	-	Simvastatin inhibits proliferation and osteolytic bone metastases of lung adenocarcinoma cells in vitro and in vivo. Its mechanism might be linked with regulating the CD44, P53, and MMP family and inactivating the MAPK/ERK signaling pathway.	[203]
Pitavastatin and fluvastatin		In vitro	Erlotinib	Statins/erlotinib combination’s induced cytotoxicity is synergistic, can overcome erlotinib resistance in K-ras-mutated NSCLC, and depends on apoptosis.	[231]
Simvastatin and Lovastatin	Melanoma and neuroblast-oma	In vitro	TRAIL sodium arsenite	Treatment of melanoma cells with statin enhanced TRAIL-induced apoptosis due to suppression of the NF-κB and STAT3-transcriptional targets (including COX-2) and downregulation of cFLIP-L (a caspase-8 inhibitor) protein levels. Moreover, co-treatment with sodium arsenite and TRAIL or simvastatin and TRAIL efficiently induced apoptotic commitment in human neuroblastoma cells.	[232]
Lovastatin, atorvastatin, simvastatin, pravastatin, and fluvastatin	Melanoma	In vitro	-	Pravastatin was the least effective cytotoxic of the five tested statins on melanoma cells. Lovastatin produces apoptosis in multiple melanoma cell lines through a geranylation-specific mechanism via caspase-dependent signaling.	[115]
Simvastatin	Head and neck	In vitro	Celecoxib	Simvastatin and celecoxib alone and in a combined treatment significantly reduced head and neck SCC viability, proliferation, and the secretion of IL-6 and IL-8.	[233]
Simvastatin		In vitro	Monocarboxylate transporter 1 (MCT1)	Simvastatin induces metabolic reprogramming in head and neck squamous cell carcinoma mice, reducing lactate production and promoting cancer sensitivity to MCT1 inhibitors.	[234]
Pitavastatin		In vitro	-	Pitavastatin activates the FOXO3a/PUMA apoptotic axis via regulation of nuclear translocation of FOXO3a through Akt/FOXO3a or AMPK/FOXO3a signaling.	[235]
Atorvastatin		In vitro and vivo	-	Atorvastatin significantly reduced the active form of RhoC in vitro and diminished cell motility, invasion, proliferation, and colony formation. A significant decrease in p-ERK1/2 and p-STAT3 in atorvastatin-treated cell lines was observed. In vivo, experiments have shown the inhibition of angiogenesis and lung metastases.	[236]

Abbreviations: GGPP, geranylgeranyl pyrophosphate; TRAIL, TNF-related apoptosis-inducing; FPP, farnesyl pyrophosphate; GPP, geranylgeranyl pyrophosphate; NSCLC, non-small cell lung cancer; VEGF, vascular endothelial growth factor; MPP, matrix metalloproteinase; SCC, squamous cell carcinoma.

### 3.5. Clinical Trials of Statins

In recent years, in light of achieving promising preclinical results, the potential anticancer effects of statins in clinical studies have been evaluated for several cancer types, either as a single agent or combined with chemotherapeutic agents used in standard treatment protocols (Table 3).

A prospective study designed to determine biomarkers of statin responsiveness in postmenopausal women at increased risk of breast cancer revealed that 24–28 weeks of use of simvastatin significantly decreased lipid profiles, high-sensitivity C-reactive protein, and estrone sulfate levels [237]. However, another study in women at high risk for breast cancer failed to prove significant modulation of the breast cancer biomarker after 6 months of lovastatin therapy [238]. Neoadjuvant fluvastatin for breast cancer patients with a high grade at a high dose (80 mg/day) reduced breast tumor proliferation and increased apoptosis compared with a lower 20 mg/day treatment [239]. Moreover, neoadjuvant fluvastatin (80 mg/day) treatment for 4–12 weeks before radical prostatectomy is associated with apoptosis induction in localized prostate cancer patients [240]. Another study found that simvastatin might improve the efficacy of gefitinib in gefitinib-resistant non-small cell lung cancer (NSCLC) patients [241]. Further, the addition of lovastatin to thalidomide and dexamethasone in the treatment of patients with relapsed or refractory multiple myeloma improved overall survival and progression-free survival [242]. A phase I clinical trial was performed to investigate the safety and to recommend the dose of rosuvastatin in patients with advanced solid malignancies, in which an escalating dose of rosuvastatin (1–8 mg/kg/day) in combination with the standard erlotinib dose was used. The combination has resulted in a disease stabilization rate of 25%; meanwhile, a high level of muscle toxicities has been detected, limiting the use of this combination [243]. In the phase II clinical trial, the safety and efficacy of simvastatin combined with cetuximab were evaluated in metastatic CRC patients with KRAS mutations who had previously received fluoropyrimidine, oxaliplatin, and irinotecan-based treatment regimens. The clinical data revealed that only 4 out of 18 (22.2%) patients were free from progression at the primary end, with 20.3 to 47 weeks of progression [244].

Overall, clinical trial findings regarding statin use in cancer have been inconclusive. Therefore, additional well-designed clinical trials are needed to confirm statins’ safe and effective use.

**Table 3:** Statin use in anticancer clinical trials.

Statins	Cancer Type	Phase	No. of Patients	Combination Agent	Findings	Ref.
Fluvastatin	Breast	II	40	3–6 months statin perioperative	After statins use, Ki67 was reduced and caspase 3 increased.	[239]
Simvastatin		III	60	FAC	Simvastatin combined with FAC shows improvements in objective response rate and pathological response in patients with LABC.	[245]
Atorvastatin		II	42	-	Atorvastatin decreased breast cancer proliferation via cell cycle regulatory effects through cyclin D1 and p27.	[246]
Pravastatin	Liver	II	83	Transcatheter arterial embolization (TAE) followed by 5-FU	Median survival was 18 months in the pravastatin group compared to 9 months in controls (*p* = 0.006).	[247]
Pravastatin		II	312	Sorafenib	A combination of sorafenib and pravastatin did not improve overall survival compared to sorafenib alone.	[248]
Simvastatin	Gastric	III		Capecitabine–cisplatin	Adding simvastatin to capecitabine–cisplatin does not increase PFS.	[249]
Pravastatin		II	30	ECC	The addition of pravastatin to ECC in patients with advanced gastric cancer did not improve the outcome.	[250]
Simvastatin	Colorectal	III	269	FOLFIRI/XELIRI	Adding simvastatin to the XELIRI/FOLFIRI regimens did not improve PFS in patients with previously treated metastatic colorectal cancer.	[251]

Abbreviations: FAC, fluorouracil, adriamycin, and cyclophosphamide; LABC, locally advanced breast cancer; PFS, progression-free survival; ECC, epirubicin, cisplatin, and capecitabine; FOLFIRI, fluorouracil, irinotecan, and leucovorin; XELIRI, capecitabine and irinotecan.

### 3.6. Drug Efflux Pumps in Cancer and Modulating Efflux Pumps in Cancer with Statins

Cancer chemoresistance is associated with tumor relapse and metastases and remains a major health challenge [252,253]. Several cellular and non-cellular pathways have been suggested as hypothetical mechanisms behind MDR, such as removing hydrophobic drugs from cells through an increased energy-dependent efflux [254,255]. ABC proteins are members of the membrane transport system superfamily; they are responsible for the translocation of numerous substrates (e.g., ions, sugars, amino acids, peptides, lipids, and xenobiotics) using energy derived from the hydrolysis of ATP [256,257]. ABC transporters are expressed naturally in diverse tissues and protect the body from xenobiotics [258,259]. Forty-nine different ABC transporter genes are found in the human genome, categorized into seven distinct subfamilies. Among them, three subfamilies commonly involved in cancer MDR are P-glycoprotein (P-gp), multidrug resistance protein 1 (MRP 1 or ABCC1), and breast cancer resistance protein (BCRP or ABCG2) [260,261].

ABCB1 transporter overexpression in cancer cells results in developing resistance to several chemotherapeutic drugs, including doxorubicin, daunorubicin, vinblastine, vincristine, docetaxel, paclitaxel, actinomycin-D, teniposide, and etoposide [262]. P-gp is the best-described efflux pump that facilitates MDR in malignancy [263]. Subcellular expression of P-gp may play a crucial role in MDR in several cancers [27,264,265,266].

MRP1 or ABCC1 is structurally related to P-gp [267]. ABCC1 transports anticancer drugs such as methotrexate, etoposide, epirubicin, doxorubicin, and vincristine [268]. Although there is high expression of ABCC1 in almost all cancer types, there has been no evidence for an association between its expression and drug resistance [269].

ABCG2, also named BCRP, is expressed in many biological tissues and numerous solid tumors as well as hematological malignancies [270]. ABCG2 transports many chemotherapeutic agents, including mitoxantrone, topotecan, SN-38, tyrosine kinase inhibitors, and doxorubicin [271].

ABC transporters are located in the lipid rafts, plasma membrane domains rich in cholesterol and glycosphingolipids. Studies have revealed that membrane cholesterol is essential in regulating the activity of ABCB1 and ABCG2; accordingly, lipid-lowering drugs can potentially overcome drug resistance [272,273]. Glodkowka-Mrowka et al. reported that statin-induced depletion of cellular cholesterol in leukemic cells inhibited ABCB1 and ABCG2 multidrug transporters [274]. Moreover, it has been published that statins inhibit the activity of ABCB1 in resistant human tumor cell lines [275]. Another study on colon cancer cells combined with statins and flavonoids showed that statins can also affect cholesterol biosynthesis by inhibiting HMG-CoA reductase. Still, they can also act through different mechanisms [276]. A similar concept has been previously implemented by Staedler et al., who demonstrated increased growth inhibitory effects of atorvastatin when combined with other cholesterol-lowering agents in human glioblastoma cells [277].

Simvastatin was found to induce a glutathione (GSH)-mediated reduction of ABCG4 levels, which is responsible for intracellular doxorubicin and cisplatin efflux, increasing the sensitivity of prostate cancer cells to both doxorubicin and cisplatin. Interestingly, simvastatin and doxorubicin combined significantly reduced tumor growth and size without clear doxorubicin-induced cardiotoxicity [278]. Another study was performed in which simvastatin and mevastatin effectively revert doxorubicin resistance in human malignant mesothelioma through inactivation of the ABC transporter P-gp via nitric oxide (NO)-dependent nitration in the tyrosine residue of P-gp, responsible for the efflux of doxorubicin [279]. Moreover, simvastatin and mevastatin, combined with flavonoids, suppressed doxorubicin resistance in colon cancer cells [280]. Simvastatin and phenothiazine derivative combinations reverse resistance to doxorubicin in colon cancer cells by suppressing the expression of ABCB1 (P-gp) and inflammation markers Cox-2 [281]. Furthermore, simvastatin-reversed doxorubicin mediates resistance in unmated chronic lymphocytic leukemia (CLL) cells via upregulating Ras/ERK1-2, RhoA/RhoA kinase, Akt, HIF-1, and P-gp activities [282]. The inhibition of drug efflux mediated by P-gp transporter in the presence of statins has been reported in human neuroblastoma cells [283]. Statins have also been revealed to bind directly to P-gp, facilitating doxorubicin transport in cancer cells [275].

As previously mentioned, repurposing statins could be a logical way to overcome cancer’s multidrug resistance.

## 4. Conclusions

Statins are HMG-CoA reductase inhibitors belonging to a class of lipid-lowering drugs originally developed to treat cardiovascular disease. In recent decades, statins have been widely recognized as pleiotropic drugs.

Since developing novel chemotherapeutic drugs requires large investments, drug repurposing offers a new approach that can provide alternatives as adjuvants in treating resistant microbial infections and progressive cancerous diseases with overexpressed efflux pumps.

There are some similarities between the effects of statins on bacteria and tumor cells. In both cases, statins can affect cell proliferation and survival by modulating key signaling pathways. Statins can inhibit the mevalonate pathway, which is important for synthesizing cholesterol and isoprenoids, leading to downstream effects on cellular processes such as cell membrane formation, signal transduction, and protein prenylation. This property can result in antitumor effects in tumor cells by reducing cell proliferation and promoting apoptosis. Similarly, statin-mediated inhibition of the mevalonate pathway in bacteria can lead to decreased bacterial growth and increased susceptibility to antibiotics. Bacteria express different subtypes of the HMG-CoA reductase enzyme, and the role of the mevalonate pathway in the antibacterial effect should be investigated in more detail. Molecular docking to study the interactions between statins (fluvastatin, cerivastatin, and rosuvastatin) and HMG-CoA reductase and to evaluate their binding efficiency with HMG-CoA reductase indicates that fluvastatin binds strongly with amino acids at the active site of HMG-CoA. In contrast, rosuvastatin has a comparatively weaker interaction [284]. On the other hand, some natural compounds, such as rutin, amentoflavone, and ganomycin I, were reported to bind to HMG-CoA reductase with high docking scores [285,286]. In silico studies were performed to reveal the possible bacterial protein targets of statins other than HMG-CoA reductase. These molecular targets are structural and enzymatic proteins essential for bacteria’s survival and proliferation. Simvastatin, rosuvastatin, and fluvastatin had good binding interactions with various microbial structures [287]. Further computer-aided drug identification of target–ligand interaction at the molecular level between statin and HMG-CoA reductase is warranted to give a deeper insight for further optimization of the statin molecules.

A similarity between the bacterial and cancer-related effects of statins is the potential for statins to modulate immune responses. In the presence of tumor cells, statins have been shown to have immunomodulatory effects by reducing inflammation and promoting antitumor immune responses. In bacterial infections, statins may also have immunomodulatory effects by enhancing host immune responses and reducing the severity of infections. The mechanisms of action may differ between tumor cells and bacteria, but there are some resemblances in how statins can provide inhibitory effects against these two types of cells.

In conclusion, statins might potentiate antibacterial and anticancer therapy, as was published in epidemiological and clinical studies. However, well-designed clinical trials are required to transfer this to patients’ standards of treatment.

## Figures and Tables

**Table 1:** Statin use in epidemiological studies.

Statins	Cancer Type	Study Type	No. of Patients	Combination Agent	Findings	Ref.
NR	Breast	Cohort study	17,880	-	The use of statins after diagnosis of breast cancer reduced breast cancer mortality and all-cause mortality.	[165]
Lipophilic statins		Cohort study	1811	-	The use of lipophilic statins after breast cancer diagnosis was related to a reduced risk of breast cancer recurrence.	[166]
Atorvastatin, rosuvastatin, simvastatin, lovastatin, pitavastatin, pravastatin, and fluvastatin		Meta-analysis	75,684	-	Lipophilic statin use was associated with improved DFS for patients with breast cancer. In addition, there was an improvement in cancer-specific survival and overall survival.	[167]
Atorvastatin, simvastatin, lovastatin, pravastatin and rosuvastatin	Prostate	Cohort study	44,126	-	Statins are associated with a lower risk of PTEN-null and lethal prostate cancer.	[168]
NR		Case–control study	1367	NSAIDs	The use of statins alone or in combination with NSAIDs had no protective effects on the risk of advanced prostate cancer.	[169]
NR	Colorectal	Meta-analysis	~8.2 million	-	The study confirmed a modest significant protective effect of statin use at therapeutic doses on CRC.	[170]
NR	Ovarian	Cohort study	4419	-	Ovarian cancer patients’ statin use after diagnosis was not associated with the reduction in mortality.	[171]
NR		Meta-analysis	1,254,501		Statin use was not significantly associated with risks but reduced mortality in ovarian cancer patients.	[172]
Atorvastatin, lovastatin, simvastatin, pravastatin, rosuvastatin and fluvastatin		Cohort study	958	-	In ovarian cancer, lipophilic statins slightly improve patients’ survival with no effect with hydrophilic statins.	[162]
Atorvastatin, rosuvastatin, simvastatin, lovastatin, pitavastatin, pravastatin, and fluvastatin	Endometrial cancer	Meta-analysis	~1,700,000	-	Statin use decreases mortality of endometrial cancer risks.	[173]
NR	Head and neck	Cohort study	1194	-	Statin use at the time of the diagnosis improved overall and disease-free survival of HPV-negative SCC of the larynx, hypopharynx, and nasopharynx.	[174]
Atorvastatin, rosuvastatin, simvastatin, pravastatin, and fluvastatin	Glioblastoma	Case–control study	27,159	-	Statin use was not associated with the risk of GBM.	[175]
Simvastatin, atorvastatin, cerivastatin, fluvastatin, lovastatin, and pravastatin		Cohort study	1093	-	The use of statins was not associated with improved OS or PFS in GBM patients.	[176]
Atorvastatin, simvastatin, lovastatin, pravastatin, and rosuvastatin	Lung	Cohort study	19,974	-	Statin use in patients with SCC and adenocarcinoma lung cancer was associated with a decreased mortality risk.	[177]

Abbreviations: NR, not reported; DFS, disease-free survival; NSAIDs, non-steroidal anti-inflammatory drugs; CRC, colorectal cancer; GBM, glioblastoma multiforme; OS, overall survival; PFS, progression-free survival; SCC, squamous cell carcinoma.

## Data Availability

Not applicable.
